# Investigating the Causal Association Between *Helicobacter pylori* Infection and Diabetic Nephropathy: A Two-Sample Mendelian Randomization Study

**DOI:** 10.1155/ije/3387412

**Published:** 2025-12-04

**Authors:** Lu Yu, Xinye Niu, Yu Zhai

**Affiliations:** ^1^Department of Endocrinology, Changzhou Hospital Affiliated to Nanjing University of Chinese Medicine, Changzhou 213003, China; ^2^Department of Orthopedics and Traumatology, Changzhou Hospital Affiliated to Nanjing University of Chinese Medicine, Changzhou 213003, China

**Keywords:** diabetic nephropathy, genome-wide association study, *Helicobacter pylori*, instrumental variables, Mendelian randomization

## Abstract

**Objective:**

Diabetic nephropathy (DN) is a leading cause of end-stage renal disease. This study investigated the potential causal association between *Helicobacter pylori* infection and DN.

**Methods:**

The two-sample Mendelian randomization (MR) methodology and public data on DN and *H. pylori* infection from genome-wide association studies (GWASs) were used. The primary MR analytical method was the inverse variance weighted (IVW), complemented by additional methods such as MR-Egger, weighted median, and weighted mode. Results were validated through extensive sensitivity analyses, including tests for pleiotropy (PhenoScanner), directionality (bidirectional MR and Steiger test), and heterogeneity. A false discovery rate (FDR) was applied to correct for multiple testing.

**Results:**

Among 7 *H. pylori* antibody markers, only genetically predicted catalase antibody levels showed a suggestive protective association with DN (odds ratio [OR] = 0.90, 95% confidence interval [CI]: 0.82–0.99, *p* = 0.03). However, this association did not withstand correction for multiple testing (P-FDR = 0.21). No significant causal effects were observed for other antibody markers. Sensitivity analyses found no evidence of horizontal pleiotropy and consistently supported a causal direction from *H. pylori* exposure to DN.

**Conclusion:**

Our findings provide suggestive evidence for a potential causal link between the host immune response to *H. pylori* catalase and a lower risk of DN. This specific, biologically plausible pathway warrants further investigation in larger, more diverse populations to confirm its potential role in the pathogenesis of DN.

## 1. Introduction

Diabetic nephropathy (DN) is characterized by renal damage due to microvascular complications from diabetes, primarily indicated by persistent proteinuria and a gradual decline in renal function [1]. DN stands as the predominant complication of Type 2 diabetes mellitus, constituting the primary contributor to end-stage renal disease globally [2]. This condition is marked by significant morbidity and mortality. DN occurs within a decade of Type 2 diabetes mellitus diagnosis in about 40% of patients [3, 4]. The epidemiological landscape of DN underscores its status as a multifaceted complication of diabetes, urgently demanding a thorough understanding of its risk factors and pathophysiological mechanisms [5, 6].

Among the potential contributors to DN, the role of *Helicobacter pylori* infection presents an intriguing area of investigation. *H. pylori* is a bacterium involved in various gastrointestinal diseases and has been hypothesized to influence the pathogenesis of diabetic complications, including nephropathy [7, 8]. Research over the years has suggested a complex relationship between *H. pylori* infection and various diabetic complications, highlighting a broader systemic influence that this bacterium might have beyond the gastrointestinal tract [9]. Indeed, *H. pylori* infection can exacerbate the inflammatory state typically associated with diabetes by influencing the immune responses and possibly altering metabolic pathways involved in glucose regulation, drawing attention to its potential role in the pathogenesis of diabetes-related complications [10, 11]. Various pathogenic mechanisms have been proposed, wherein *H. pylori* may exacerbate DN progression. For instance, the bacterium's ability to induce systemic inflammation could enhance inflammatory processes within the kidney via cytokines such as Interleukin (IL) 6 and tumor necrosis factor (TNF)-α, which are known to be elevated in *H. pylori* infections [12]. In addition, *H. pylori* may contribute to kidney fibrosis through increased tumor growth factor (TGF)-β, a key fibrotic mediator, and possibly impact glucose metabolism by altering systemic metabolic processes, further impairing glycemic control in diabetic patients. Despite these associations, the causal relationship between *H. pylori* infection and DN remains controversial, with some studies suggesting potential coincidental associations due to confounding factors.

Mendelian randomization (MR) is an innovative epidemiological method that uses genetic variants as instrumental variables (IVs) to determine causal associations between modifiable risk factors and health outcomes, effectively sidestepping the confounders and reverse causation common in observational studies [13]. By exploiting the random assignment of alleles at conception, akin to a natural randomized controlled trial, MR can robustly infer causality from the genetic predisposition to certain exposures, such as biomarker levels or pathogen presence, and their effects on diseases [14]. This approach utilizes genetic data from genome-wide association studies (GWASs) to explore various exposures and their causal impact on health outcomes, promising significant advancements in uncovering complex etiological pathways. MR's strength lies in its potential to guide future studies on preventive strategies and therapeutic interventions based on causal evidence [15]. MR stands for a powerful tool in epidemiology, offering novel insights into disease causation and pinpointing targets for future public health and clinical strategies [16]. This approach could enable researchers to infer causality from the directional association between genetically determined *H. pylori* infection and DN risk.

Given the inconclusive findings in previous studies, this study aimed to explore the genetically causal relationship between DN and *H. pylori* using the MR methodology. This effort aims to provide stronger evidence for the prevention and management of DN, thereby improving clinical risk assessment and treatment strategies.

## 2. Methods

### 2.1. Study Design

A two-sample MR study utilizing summary-level data from large-scale genetic databases was designed to investigate the causal relationship between *H. pylori* infection and DN. The present MR analysis follows the three core principles of MR: relevance, independence, and exclusion-restriction [17]. Firstly, genetic variants strongly associated with *H. pylori* antibody levels were selected as IVs to satisfy the relevance criterion. These IVs were chosen based on their significant association with *H. pylori* infection, ensuring a robust and direct relationship with the exposure. It was verified that the selected IVs for *H. pylori* infection were not associated with known risk factors for DN, apart from their effect on *H. pylori* infection levels, to address the independence criterion and minimize confounding. This step aims to reduce the potential for confounded associations that could bias our causal inference. For the exclusion-restriction principle, methods such as MR-Egger regression were employed to detect and adjust for potential pleiotropic effects where the genetic variants might influence DN through pathways other than *H. pylori* infection. This approach helps verify that the genetic variants impact the risk of DN solely through their effect on *H. pylori* infection levels.

### 2.2. Data Sources

Exposure to *H. pylori* infection was proxied through various antibody measures, including anti-*H. pylori* IgG, GroEL, OMP, urea, VacA, catalase, and CagA. Specifically, the exposure data encompass 2251 to 4683 cases for the different *H. pylori* antibody measures. These exposure datasets are derived from the MRC-IEU database (available at https://gwas.mrcieu.ac.uk/) [18], providing a comprehensive genetic instrument selection for *H. pylori* infection. The GWAS of DN includes 3283 cases out of 210,463 individuals, accompanied by 16,380,453 associated single nucleotide polymorphisms (SNPs), and was sourced from the FinnGen database (available at https://www.finngen.fi/en) [19]. Detailed information on the exposure and outcome data, corresponding SNP counts, and case numbers can be found in Table 1.

### 2.3. Selection of IVs

A rigorous selection protocol was implemented for IVs to ensure their integrity and effectiveness in investigating the causal relationship between *H. pylori* infection and the development of DN. Initially, SNPs significantly associated with *H. pylori* infection were identified, requiring a *p* value < 5 × 10^−6^ to guarantee the selection of SNPs with strong statistical relevance. SNPs with a minor allele frequency (MAF) of > 0.01 were focused on, ensuring the inclusion of common genetic variants and enhancing the study's reliability. In order to avoid bias from linkage disequilibrium (LD), SNPs within a 10,000-kb radius with an *R*^2^ < 0.001 were excluded to maintain the independence of the IVs. Proxy SNPs with high LD (*R*^2^ > 0.8) with the original IVs were used when necessary to ensure their comprehensive representation in the analysis. Finally, the strength of each SNP as an IV was evaluated by calculating its *F*-statistic (*F* = *R*^2^ × (*N*−2)/(1−*R*^2^)). To ensure the robustness of our instruments, we confirmed that all selected SNPs met the threshold of *F* > 10. We have calculated the F-statistics for each set of IVs, and their mean, range, and minimum values are now reported in Table S1. Crucially, the minimum *F*-statistic across all exposures exceeded 10, indicating that weak instrument bias is unlikely in our study.

### 2.4. Statistical Analysis

In the MR analysis, four distinct methods were applied to estimate and validate the causal associations: inverse variance weighted (IVW), MR Egger, weighted median, and weighted mode. The IVW method served as the primary basis for the interpretation of the results [20–22]. To test the directionality of the observed causal association, we performed two additional analyses. First, we conducted a bidirectional MR analysis, treating DN as the exposure and *H. pylori* antibody levels as the outcome. Second, we applied the Steiger directionality test to infer the most likely causal direction for each significant association [23]. Furthermore, to account for multiple testing across different *H. pylori* antibody exposures, we applied a false discovery rate (FDR) correction using the Benjamini–Hochberg method. An FDR-adjusted *p* value < 0.05 was considered statistically significant.

### 2.5. Sensitivity Analyses

Sensitivity analyses were conducted to address the potential presence of pleiotropy within the MR analysis. Heterogeneity among the IVs was assessed using Cochran's *Q* test, where a *p* value > 0.05 was indicative of low heterogeneity, suggesting that the variations in the estimates provided by the IVs were random and had minimal impact on the IVW results [24]. MR-Egger regression was employed to evaluate the influence of pleiotropy on the association estimates [25]. The approach of MR-Egger allows for the exploration of horizontal pleiotropy, with an intercept term close to zero or statistically nonsignificant, indicating an absence of pleiotropic effects. The leave-one-out approach was applied to complement the sensitivity analyses [26]. To further systematically assess potential violations of the independence assumption, we utilized the PhenoScanner V2 database [27]. All IVs that were significantly associated with *H. pylori* catalase antibody levels were queried to check for any previously reported associations with potential confounders of DN (e.g., obesity, hypertension, and dyslipidemia).

All analyses in this study were conducted using the “TwoSampleMR” package in *R*, facilitating the integration and comparison of summary data from different sources for the MR analysis. For visualization purposes, scatter plots and sensitivity analysis plots were generated to illustrate the genetic associations and assess the robustness of the causal inferences, respectively.

## 3. Results

### 3.1. Selection of IVs

In this study, IVs were selected to investigate the causal association between *H. pylori* infection and DN. The exposure variables were *H. pylori* IgG, GroEL, OMP, urea, VacA, catalase, and CagA levels. The relevant IVs identified for each antibody were 12 for IgG, five for GroEL, 10 for OMP, 10 for urea, 15 for VacA, nine for catalase, and 15 for CagA. For the OMP antibody levels, rs12550507 was used as a proxy for rs60949128, and for VacA antibody levels, rs78230473 was used as a proxy for rs148556020. The mean F-statistics for the IVs were 18.64 for IgG, 13.49 for GroEL, 19.51 for OMP, 19.24 for urea, 19.96 for VacA, 17.69 for catalase, and 19.24 for CagA, indicating strong instrument strength across all exposures (Table S1).

### 3.2. MR Analysis

**Figure 1:**
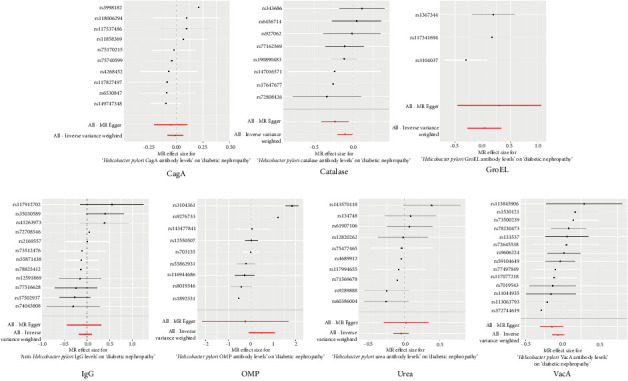
Forest plot of the causal association between *Helicobacter pylori* infection (proxied by antibody levels) and the risk of diabetic nephropathy. This plot visualizes the odds ratios for the causal effect of different *H. pylori* antibody exposures on diabetic nephropathy, as estimated by various MR methods. It allows for a quick comparison of the consistency and magnitude of effects across different analytical approaches.

**Figure 2:**
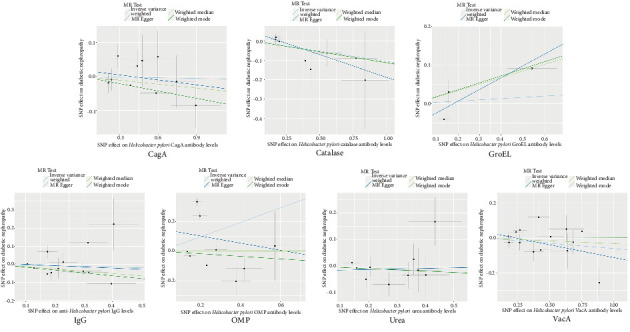
Scatter plot of instrumental variable effects on *Helicobacter pylori* catalase antibody levels versus their effects on diabetic nephropathy. This plot illustrates the relationship between the SNP effects on the exposure (*H. pylori* catalase antibodies) and the SNP effects on the outcome (diabetic nephropathy). The slope of the regression line for each method represents the estimated causal effect.

The results across multiple methods did not consistently indicate a statistically significant association between *H. pylori* antibody levels and the risk of DN (Figures 1 and 2, Table S2). The analysis of catalase antibody levels suggested a potential causal association with DN (IVW, OR = 0.90 95% CI: 0.82–0.99, *p* = 0.03), supported by the MR-Egger (*p* = 0.04), and raw (*p* = 0.05) methods. However, after applying the FDR correction for multiple testing, the association between catalase antibody levels and DN was no longer statistically significant (P-FDR = 0.21, Table S2).

No significant causal associations were observed for OMP antibody levels (IVW, OR = 1.63, 95% CI: 0.91–2.93, *p* = 0.1), *H. pylori* IgG levels (IVW, OR = 0.96, 95% CI: 0.83–1.11, *p* = 0.61), *H. pylori* GroEL antibody levels (IVW, OR = 1.03, 95% CI: 0.76–1.4, *p* = 0.84), urea (IVW, OR = 0.95, 95% CI: 0.85–1.06, *p* = 0.39), VacA antibody levels (IVW, OR = 0.94, 95% CI: 0.87–1.02, *p* = 0.15), or CagA antibody levels (OR = 0.99, 95% CI: 0.92–1.07, *p* = 0.86).

### 3.3. Sensitivity Testing

**Figure 3:**
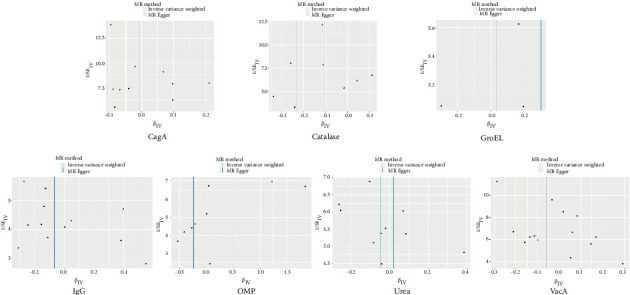
Funnel plot for assessing pleiotropy in the MR analysis of *Helicobacter pylori* catalase antibody levels and diabetic nephropathy. This plot assesses the potential for heterogeneity and horizontal pleiotropy in the analysis of the catalase exposure. A symmetrical distribution of SNPs around the causal estimate suggests an absence of significant pleiotropy, whereas asymmetry may indicate its presence.

**Figure 4:**
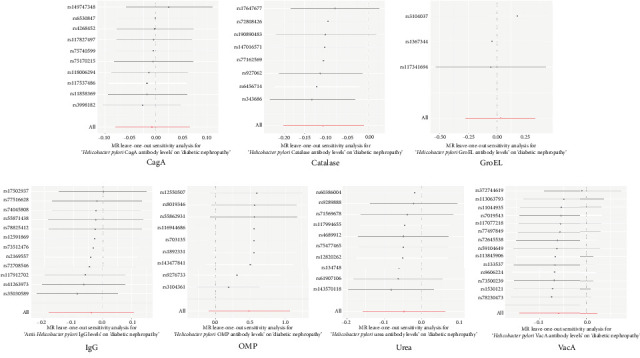
Leave-one-out sensitivity analysis for the causal effect of *Helicobacter pylori* catalase antibody levels on diabetic nephropathy. This analysis iteratively removes one SNP at a time and recalculates the causal estimate to determine if any single SNP is disproportionately influencing the overall result. The consistency of estimates supports the robustness of the finding.

The Steiger directionality test supported a causal direction from *H. pylori* antibody levels to DN for all tested associations (all *p* < 0.05, Table S3). The analysis revealed low heterogeneity for *H. pylori* IgG, GroEL, urea, VacA, catalase, and CagA antibody levels, with leave-one-out and *Q* statistics indicating no significant dispersion (*p* > 0.05 for all but OMP) (Figures 3 and 4, Table S4). MR-Egger intercepts suggested an absence of horizontal pleiotropy, affirming the reliability of these causal estimates. Nevertheless, significant heterogeneity was noted for OMP antibody levels (*Q* = 174.821, *p* < 0.001), and as the IVW results were the main outcome, the heterogeneity was acceptable (Table S4). In the MR-PRESSO analyses, adjusting for detected outliers confirmed that pleiotropy did not significantly affect the results (Table S5). Our query of the PhenoScanner database revealed that our IVs for catalase antibody levels were not significantly associated with any known risk factors or potential confounding phenotypes for DN (Table S6). This finding provides further support for the validity of our instruments and strengthens the inference of a direct causal effect.

### 3.4. Bidirectional MR Analysis

To rigorously test for reverse causality, we conducted a bidirectional MR analysis, treating DN as the exposure and *H. pylori* antibody levels as outcomes. Our results showed no significant causal effect of genetically predicted DN on any of the *H. pylori* antibody levels (all *p* > 0.05, Table S7). While we observed some heterogeneity for the effect on CagA antibody levels (*p* < 0.05, Table S8), sensitivity analyses consistently indicated no evidence of horizontal pleiotropy across all reverse associations (MR-Egger intercept *p* > 0.05, Table S8; MR-PRESSO *p* > 0.05, Table S9). Collectively, these findings robustly support our primary hypothesis that the causal arrow points from *H. pylori* infection to DN, not the other way around.

## 4. Discussion

The present MR analysis comprehensively evaluated the possible causal associations between multiple antibody levels representing *H. pylori* and DN, employing various MR methods to ensure robustness and reliability in our causal inference. The present study suggested a causal association between catalase antibody levels and DN. In contrast, the other markers of *H. pylori* infection (OMP antibody, *H. pylori* IgG, *H. pylori* GroEL antibody, urea, VacA antibody, or CagA antibody) showed no statistically significant causal associations.

In this study, we employed a multiantigen approach to comprehensively evaluate the host's immunological response to various *H. pylori* components. This strategy allows for a broader assessment of the relationship between the infection and DN, rather than relying on a single marker such as general IgG levels [28]. The unique causal association observed with catalase antibody levels warrants a specific explanation, which may lie in its distinct immunological and mechanistic properties. Firstly, catalase is a highly immunogenic antigen of *H. pylori*, capable of inducing a strong and specific immune response in the host [29]. This high immunogenicity might lead to a more robust and detectable antibody signature compared to other antigens. Secondly, and more critically, catalase plays a direct role in a pathogenic mechanism highly relevant to DN [30]. Its primary function is to neutralize hydrogen peroxide, protecting the bacterium from oxidative stress generated by host immune cells in the gastric mucosa [31]. Oxidative stress is also a core pathological driver in the progression of DN [32]. Therefore, a host immune response specifically targeting catalase may not only signify an *H. pylori* infection but could also uniquely reflect a host–pathogen interaction directly linked to the oxidative stress pathways central to DN pathogenesis. This provides a compelling biological rationale for the specific association we observed.

*H. pylori* infection is known for its association with gastric diseases, such as peptic ulcers and gastric cancer [33–35]. However, recent studies have expanded our understanding of *H. pylori*'s influence, suggesting its involvement in several extragastric diseases, possibly through chronic systemic inflammation and molecular mimicry [36–38]. The systemic inflammation triggered by *H. pylori* can potentially interfere with normal physiological processes, becoming a risk factor for atherosclerosis, insulin resistance, and other conditions that share common pathways with diabetic complications [39].

The mechanisms underlying DN are multifaceted, involving hemodynamic changes, metabolic pathways, and inflammatory responses. Key pathways involved in DN include the renin-angiotensin system, reactive oxygen species production, and advanced glycation end-products, among others [40–42]. Given *H. pylori*'s role in promoting systemic inflammation, it is reasonable to hypothesize that *H. pylori* infection could exacerbate these underlying mechanisms contributing to DN [43–45]. Specifically, *H. pylori*–induced systemic inflammation might amplify kidney inflammatory responses, accelerating DN progression. Moreover, molecular mimicry between *H. pylori* and host structures can induce antibody production that may be autoreactive, contributing to autoimmune responses seen in other extragastric manifestations. This phenomenon could potentially play a role in the pathogenesis of DN, where autoimmune mechanisms have been proposed as contributing factors [46, 47].

A unique immunological profile exists when evaluating the serological landscape of *H. pylori* infection in relation to DN using MR. Different biomarkers were used to represent *H. pylori* exposure, including *H. pylori* IgG, GroEL, OMP, urea, VacA, CagA, and catalase antibodies. Among these, only the presence of catalase antibodies was found to have a causal association with DN. This outcome necessitates a multifaceted interpretation. Indeed, the catalase in *H. pylori* has a high level of immunogenicity, which means that it can induce a strong immune response in the host [48, 49]. This strong immune response could be reflected in the higher prevalence of antibodies against the catalase compared with other antigens [50]. The immune activation by catalase might be more specific and directly associated with pathways that exacerbate DN. Unlike other *H. pylori* antigens that might trigger broader immune responses, catalase could be influencing specific inflammatory or immune pathways that are particularly relevant to the pathophysiology of DN. This specificity might explain why only catalase antibodies have shown a causal relationship in the present study.

Meanwhile, other biomarkers such as *H. pylori* IgG, GroEL, OMP, urea, VacA, and CagA showed no causal associations with DN. Several factors could explain why these other antigens did not exhibit a similar association. Firstly, these antigens might have lower immunogenicity than catalase, leading to weaker immune responses and less detectable antibody levels [51, 52]. Secondly, variability in antigen expression among different *H. pylori* strains could mean that not all antigens are expressed sufficiently to trigger a detectable immune response. Furthermore, it is possible that these antigens do not directly impact the pathophysiological pathways critical to DN development, unlike catalase, which may influence disease progression through its role in managing oxidative stress and inflammation [53].

A comprehensive meta-analysis involving over 20,000 participants across 39 studies found a significant association between *H. pylori* infection and diabetes mellitus and DN. Specifically, the study revealed that *H. pylori* infection is associated with an increased risk of developing diabetes mellitus, with odds ratios indicating a stronger association for Type 2 diabetes compared with Type 1 [54]. Chua et al. conducted a meta-analysis to explore the association between Type 1 diabetes and *H. pylori* infection, finding a significant association. It suggests that *H. pylori* could be a factor in the pathogenesis of Type 1 diabetes, highlighting the importance of considering this infection in Type 1 diabetes management and studies [8]. Shi et al. reviewed and analyzed the association between *H. pylori* infection and proteinuria in Type 2 diabetes [55]. Their findings indicate an association between *H. pylori* infection and the occurrence of proteinuria, suggesting *H. pylori*'s role in kidney complications in patients with Type 2 diabetes, consistent with a meta-analysis [56]. Zhou et al. analyzed 241 patients with Type 2 diabetes and 69 nondiabetic subjects with dyspeptic symptoms and reported that patients with DN were more susceptible to *H. pylori* infection [57]. These findings suggest that *H. pylori* plays a pathogenic role throughout the progression from disease-free status to diabetes mellitus and subsequently to DN, particularly in patients with Type 2 diabetes.

The core strength of the present study lies in the application of MR to dissect the causal relationship between *H. pylori* infection and DN, leveraging genetic variations as IVs. Observational studies are susceptible to confounding, where factors related to the exposure (*H. pylori* infection) and the outcome (DN) might influence the observed association. For instance, socioeconomic status, lifestyle factors, and other comorbidities could confound the relationships, making them appear stronger or weaker than they are [57]. While MR studies significantly minimize the confounding and biases inherent in observational studies, they provide a more refined insight into the genetic underpinnings of disease associations [58]. Observational studies often rely on self-reported data or clinical records for exposure and outcome information, which can introduce measurement bias. Inaccuracies in diagnosing *H. pylori* infection or the classification of DN status could affect the true association. MR studies use genetic variants as proxies for *H. pylori* infection, which are precisely measured and not subject to the same types of measurement bias that can affect observational study findings. The rigorous sensitivity analyses further reinforce the robustness of the findings by thoroughly evaluating the potential impact of pleiotropy, thus enhancing the reliability of our causal conclusions.

Nevertheless, the association or lack of association observed here could be affected by several limitations, reflecting the complex interplay between genetic predisposition and environmental factors in disease etiology. Firstly, the genetic instruments utilized primarily originate from studies in European populations, potentially limiting the generalizability of the results across diverse ethnic backgrounds due to varying genetic architectures and environmental exposures. Secondly, despite MR's strengths in clarifying genetic contributions to disease risk, it does not fully represent the myriad environmental and lifestyle factors contributing to DN's development.

## 5. Conclusions

The present MR study suggests a genetically causal association between *H. pylori* infection and DN, underscoring the potential for catalase antibodies to serve as biomarkers for screening DN in patients with *H. pylori* in the future.

## Figures and Tables

**Table 1:** Data sources.

	Trait	GWAS ID	Cases	SNPs
Exposure	*Helicobacter pylori* IgG levels	Ieu-b-4905	4683	7,247,045
	*H. pylori* GroEL antibody levels	ebi-a-GCST90006913	2716	9,172,299
	*H. pylori* OMP antibody levels	ebi-a-GCST90006914	2640	9,167,440
	*H. pylori* urea antibody levels	ebi-a-GCST90006915	2251	9,170,248
	*H. pylori* VacA antibody levels	ebi-a-GCST90006916	1571	9,178,635
	*H. pylori* catalase antibody levels	ebi-a-GCST90006912	1558	9,167,570
	*H. pylori* CagA antibody levels	ebi-a-GCST90006911	985	9,165,056
Outcome	Diabetic nephropathy	finn-b-DM_NEPHROPATHY	3283	16,380,453

## Data Availability

All data generated or analyzed during this study are included within this published article.

## Supporting Information

Supporting Information 1 Table S1: Details of SNPs after matching and their effects on exposure and outcomes. This table includes detailed information for each instrumental variable, including their F-statistics (mean, range, and minimum value) to confirm instrument strength.

Supporting Information 2 Table S2: Mendelian randomization analysis results With FDR correction. This table shows the main forward-direction causal estimates for the effect of each *H. pylori* antibody level on diabetic nephropathy, with *p* values adjusted for multiple comparisons using the FDR method.

Supporting Information 3 Table S3: Results of the Steiger directionality test. This table shows the results of the Steiger filtering analysis used to infer the causal direction between *H. pylori* antibody levels and diabetic nephropathy.

Supporting Information 4 Table S4: Heterogeneity and directional pleiotropy tests for the forward MR analysis. This table presents the detailed results of Cochran's *Q* statistic for heterogeneity and the MR-Egger intercept test for directional pleiotropy for the forward causal analysis.

Supporting Information 5 Table S5: MR-PRESSO outlier test results for the forward MR analysis. This table provides the detailed output of the MR-PRESSO analysis, including detected outliers and corrected estimates, for the forward causal analysis.

Supporting Information 6 Table S6: Results of PhenoScanner analysis for instrumental variables associated with *H. pylori* catalase antibody levels. This table lists the results from querying the PhenoScanner database to check for potential pleiotropic associations of the genetic instruments for catalase antibody levels with other traits.

Supporting Information 7 Table S7: Bidirectional Mendelian randomization analysis results with FDR correction. This table presents the main findings from the reverse MR analysis, testing the causal effect of diabetic nephropathy (as the exposure) on *H. pylori* antibody levels (as the outcome).

Supporting Information 8 Table S8: Heterogeneity and directional pleiotropy tests for the bidirectional MR analysis. This table presents the detailed results of Cochran's *Q* statistic and the MR-Egger intercept test for the reverse causal analysis.

Supporting Information 9 Table S9: MR-PRESSO outlier test results for the bidirectional MR analysis. This table provides the detailed output of the MR-PRESSO analysis for the reverse causal analysis.
